# President’s Address

**Published:** 1885-01

**Authors:** 


### President's Address.

BEFORE THE NORTHWESTERN DENTAL ASSOCIATION.

*Gentlemen of the Northwestern Dental Association:*

If I would follow a time-honored custom, in addressing you to-day, I would feel it necessary to recite to you, partially at least--the history of our profession; believing, however, that to us at present there are matters of more importance, than simply refreshing our minds with the results of the practice of by-gone years, you will permit me to depart from that appropriate custom.

Within the last few years our profession has reached an epoch when it should rise above the position it occupied in the past, and for this end some of the foremost and honored members of our profession have ardently labored, and it is but right that we all should assist in the great cause of raising the standard of our profession. As American dentists, we stand, as yet abroad as well as at home at the head of dentistry, in no country has our profession developed such an amount of inventive genius, scholarly attainment, and operative success, as in the United States; no country has taken such an advanced position as this, in so far as the facilities for teaching are concerned; all our large cities, from the Atlantic to the Pacific, and some of our best universities, have recognized our profession, by establishing schools for the special teaching of our branch; and nowhere is a dentist who is generally, and as he always ought to be a gentleman, so thoroughly recognized in the social world as in ours. Recent occurrences also show the position we occupy, and which we fully deserve, so far as the medical profession is concerned, having been recognized by that body as a specialty thereof.

Notwithstanding all these bright gems in our diadem of success, there are still blots on our escutcheon which must be fully effaced, if we expect our prospects to brighten, our reputation to grow,

And our name
To shine on the highest round of the
Ladder of fame.

Our schools, though numerous they be, though the good they have done is immeasurable, have nevertheless outgrown the present necessities, the entrance into the practice of the profession, by incompetent persons, is yet too easy a task; the lines in our schools must be drawn still closer. In correspondence with dentists practicing abroad, I find that this country cannot in the future retain the first rank as a dental educator, unless more stringent examinations are held; the idea that graduation is easy, is becoming so universally taken as a matter of fact, that, in some localities, a quack of any sort is referred to as an "American dentist." More stringent laws to govern established schools and to limit their powers, or to increase the rights and privileges of State Examining Boards, are urgently needed; the fact of so many "sham" colleges, which have existed in our country in the past years, has led to the above result. The aspect, so far as legislation is concerned, is more favorable, and to keep pace with similar movements in foreign countries, must continue so uninterruptedly; at present about twenty States have laws in full force to regulate dental practice; the results of these laws, on the whole, are quite wholesome for the East, but more or less detrimental for the West; our Northwest is sufficiently advanced to *need* laws of this character; and it is my hope and wish that Dakota may carry off the honor of being the first Territory in the Union which will fall in line with her score of full-grown sisters, the dental law States of our country, and I also hope that this Association will see fit to take steps in the right direction towards obtaining this result.

"A Vermont storekeeper set a spring gun in his store for twenty-two years without bagging anything until the other night when the old musket fell down and shot him through both legs."

